# Editorial: Bile acids in obesity-related diseases

**DOI:** 10.3389/fendo.2024.1518604

**Published:** 2024-12-23

**Authors:** Yun Shen, Xiaojiao Zheng, Ying Hu, Youping Deng, Qin Xiong

**Affiliations:** ^1^ Chronic Disease Epidemiology, Pennington Biomedical Research Center, Baton Rouge, LA, United States; ^2^ Center for Translational Medicine, Shanghai Jiao Tong University Affiliated Sixth People’s Hospital, Shanghai, China; ^3^ Department of Medical Pathology & Laboratory Medicine, University of California, Davis, Davis, CA, United States; ^4^ Department of Quantitative Health Sciences, John A. Burns School of Medicine (JABSOM) University of Hawaii at Manoa, Honolulu, HI, United States; ^5^ Department of endocrinology and metabolism, First affiliated hospital of Nanchang University, Nanchang, China

**Keywords:** bile acid, obesity, editorial, metabolism, metabolic pathway

Bile acids (BAs), traditionally recognized for their role in dietary fat digestion and cholesterol metabolism, are increasingly implicated as key regulators in obesity-related diseases (1). Beyond their classical function in the gastrointestinal tract, BAs act as signaling molecules that influence a range of metabolic pathways, including glucose homeostasis, lipid metabolism, and energy expenditure (2, 3). Obesity significantly alters BAs composition and circulation, leading to metabolic dysregulation that contributes to the pathogenesis of various conditions, including type 2 diabetes mellitus (T2DM), metabolic-associated steatotic liver disease (MASLD), and cardiovascular diseases (4–6). These changes can modulate gut microbiota composition and influence inflammatory pathways, establishing a complex network where BAs act as critical mediators of metabolic health. In this Research Topic entitled “*Bile Acids in Obesity-Related Diseases*” in Frontiers in Endocrinology, we brought 5 original articles on this increasingly important Research Topic.

The first study by Dong et al. investigated the effects of corn silk polysaccharides (CSPs) on diabetic nephropathy (DN) by using a rat model. It evaluated CSPs’ therapeutic potential through various indicators, including behavioral, histopathological, and biochemical assessments. The research focused on the interactions between CSPs, gut microbiota, and the host, utilizing a gut microbiota metabolomics approach to elucidate mechanisms that might mitigate DN. Findings revealed that CSPs significantly influence metabolic pathways related to glycerophosphate, fatty acid, BA, tyrosine, tryptophan, and phenylalanine, alongside changes in specific gut microbiota groups. These alterations suggested that CSPs may improve DN by modifying metabolite profiles and gut microbiota characteristics, thus addressing kidney inflammation and damage. The study highlighted CSPs’ potential as a safe and natural intervention for DN prevention and treatment.

Dai et al. explored the relationship among gut microbiota, metabolites and neuroendocrine during the menopausal transition. The researchers identified significant differences between pre- and post-menopausal rats in gut microbiota and metabolites related to steroid hormone biosynthesis, primary bile acid synthesis and ovarian steroidogenesis. They hypothesized that specific gut microbial composition changes could regulate BAs, which acted as signaling molecules in the hypothalamus, ultimately triggering neuroendocrine changes of menopausal transition. These findings provided a new perspective for our understanding of the physiological mechanisms underlying menopausal transition, suggesting that gut microbiota and BAs might offer new targets for future interventions and treatments.

Yin et al. investigated the metabolic changes associated with intrahepatic cholestasis of pregnancy (ICP) by comparing maternal plasma and hair metabolomes between ICP cases and healthy pregnancies. Significant differences were found in three plasma metabolites and 21 hair metabolites between the two groups. Metabolic pathway analysis of hair samples revealed 32 significantly affected pathways, including those related to glutathione metabolism and ATP-binding cassette transporters, while no significant pathway changes were observed in plasma. This study suggested that metabolomic profiling could act as a diagnostic tool for ICP. Compared to plasma, hair exhibited more distinctive metabolic profiles, and had a greater capacity to distinguish ICP from normal pregnancy. Analysis of hair metabolomes offered a fresh perspective on the pathophysiological changes associated with ICP.

Liu et al. developed risk prediction models for peripheral vascular disease (PVD) in patients with T2DM. This study revealed that total bile acids (TBA) were one of the top four predictors of PVD in T2DM, and patients with higher TBA levels were more prone to developing PVD. This article inspires the monitoring of TBA as it is beneficial for the early identification and intervention of PVD. Further research exploring the specific mechanisms by which TBA affect blood vessels will help broaden our understanding of the pathological mechanisms underlying diabetic vascular complications, facilitating better prevention and treatment of these complications.

The study by Cheng et al. provided supplementary information on the role of BAs in carotid atherosclerosis (CAS). They revealed that various BAs demonstrates distinct impacts on CAS, where some offered protective benefits, while others were independent risk factors. Additionally, the article offered predictions regarding the potential targets and pathways through which these BAs might exert their effects. These findings provided insights into potential therapeutic strategies targeting BAs for atherosclerosis treatment. The application of machine learning models shed light on developing personalized medical approaches.

In conclusion, this Research Topic of studies highlighted the multifaceted role of BAs in pathophysiology and potential treatment of various obesity-related diseases. Bile acids, beyond their traditional role in fat digestion, function as critical regulators influencing metabolic pathways that impact conditions such as DN, menopausal neuroendocrine changes, ICP, PVD and atherosclerosis. Collectively, these findings emphasized the therapeutic and diagnostic significance of BAs and related metabolic pathways, opening avenues for future research and clinical applications targeting obesity-related diseases.
